# Women in academic nephrology: have we bridged the gender gap?

**DOI:** 10.1093/ckj/sfaf019

**Published:** 2025-01-28

**Authors:** Rose Mary Attieh, Karima Wehbe, Wafaa Khaled, Darshil Jhaveri, Hay Me Me, Salman Bhutta, Kiran Munir, Kenar D Jhaveri

**Affiliations:** Division of Kidney Diseases and Hypertension, Department of Medicine, Glomerular Center at Northwell Health, Donald and Barbara Zucker School of Medicine at Hofstra/Northwell, Great Neck, NY, USA; Department of Transplant, Mayo Clinic, Jacksonville, FL, USA; Division of Nephrology and Hypertension, American University of Beirut, Beirut, Lebanon; Division of Nephrology and Hypertension, American University of Beirut, Beirut, Lebanon; Herricks High School, New Hyde Park, NY, USA; Division of Nephrology, Mayo Clinic, Phoenix, AZ, USA; Division of Kidney Diseases and Hypertension, Department of Medicine, Glomerular Center at Northwell Health, Donald and Barbara Zucker School of Medicine at Hofstra/Northwell, Great Neck, NY, USA; Division of Kidney Diseases and Hypertension, Department of Medicine, Glomerular Center at Northwell Health, Donald and Barbara Zucker School of Medicine at Hofstra/Northwell, Great Neck, NY, USA; Division of Kidney Diseases and Hypertension, Department of Medicine, Glomerular Center at Northwell Health, Donald and Barbara Zucker School of Medicine at Hofstra/Northwell, Great Neck, NY, USA

To the Editor,

Gender inequity remains deeply rooted within the academic medical community, marked by significant female underrepresentation [1, 2]. In the field of nephrology in particular, several organizations worldwide have been founded with the goal of bolstering career development opportunities for women [3]. However, whether these efforts have effectively narrowed the gender gap remains uncertain. We aimed to assess the current state of research leadership among women within nephrology subspecialties by examining vital benchmarks such as authorship of original investigations and the acquisition of extramural funding.

Abstracts accepted for oral presentation at the American Society of Nephrology (ASN) Kidney Week meetings 2017–2020 (*n* = 1401) were reviewed to capture impactful nephrology research. A subset focusing on the subspecialties of glomerular diseases (GN), transplant, and onconephrology was selected (*n* = 336). These subspecialties were chosen due to their novel nature and the significant amount of growth and cutting-edge research they have witnessed over the past decade, with numerous academic medical centers striving to enhance their expertise in these areas. The gender of the first and the last author was recorded. Abstracts were divided into two groups based on whether either the first or last author (FLA) was female, or whether both authors were male. Public databases were interrogated to find corresponding articles and the number of citations per article was analyzed for academic influence. In addition, we explored gender disparities in the size of National Institutes of Health (NIH) funding for nephrology (2017–2020) using Principal Investigator (PI) database records across the 40 top-funded academic hospitals. Gender was implied by physical appearance from institutional or social media pictures, names, and pronouns. Detailed methods are available in Supplementary Material S1.

Most oral abstracts covered GN topics (57.1%), followed by transplant (36.9%) and onconephrology (6%). Most were authored in North America (53.4%), with Europe in second place (25%). 170 abstracts (50.6%) fell in the “male-only FLA” group and 166 abstracts (49.4%) fell in the “at least one female FLA” group. Slightly more of abstracts in the latter group (51.8%) had female first authors but not last authors (Supplementary Table S2). 75.9% of abstracts (*n* = 129) with male-only FLAs had corresponding published articles, out of which 89.1% (*n* = 115) retained male-only FLAs on the full manuscript. Conversely, abstracts with at least one female FLA exhibited a higher overall publication rate of 84.9% (*n* = 140), out of which 91.4% (*n* = 128) retained at least one female FLA on the full manuscript. The chi-squared contingency test therefore showed a very strong correlation between the FLA gender groups on the abstract and the article (*P* < .001).

We found no significant inter-group difference in any of the following: the average number of abstracts having corresponding articles with FLA of the same gender group (*P *= .32), average number of abstracts having corresponding articles with FLAs of the opposite gender group (*P *= .85), and average number of abstracts that did not result in a full manuscript (*P *= 0.35). The difference between FLA gender groups remained insignificant when abstracts were stratified by sub-specialty (Supplementary Table S3). Importantly, females were first authors in only 50% of the articles falling in the “at least one female FLA” group and last authors in the remaining 50% of cases. There was no difference in the median number of citations per article between the two FLA gender groups [95% confidence interval (CI) (−5;5); *P* = .97], even when stratified by specialty, geographic location, or year (Table 1).

**Table 1:** Differences in median number of citations per article between FLA gender groups.

	Male-only FLA on article	At least one female FLA on article	Median difference in citations^a^ (range)	95% Confidence interval^b^	*P* value^b^
Total number of published articles	115	128			
Distribution by author type					
-Female first author		64 (50)			
-Female last author		25 (19.5)			
-Female first and last author		39 (30.5)			
Total number of citations	5794	6257			
Median number of citations per article	19	23	0 (−610; 594)	[−5; 5]	.97
Median number of citations per article, stratified by specialty					
Transplant	22.5	15	−5 (−312; 362)	[−1; 2]	.17
GN	19	28.5	5 (−610; 593)	[−3; 14]	.21
Onconephrology	20	65.5	22.5 (−295; 116)	[−34; 80]	.34
Median number of citations per article, stratified by geographic location					
North America	19	20	−1 (−322; 362)	[−8; 6]	.71
Europe	20.5	19	−2 (−149; 238)	[−13; 7]	.67
Asia	19	34	9 (−113; 62)	[−12; 27]	.28
Australia	101	23	−78 (−100; 91)	[−78; 91]	.71
International	21	66	26.5 (−608; 593)	[−6; 70]	.24
Median number of citations per article, stratified by year					
2017	20.5	27	3 (−162; 362)	[−6; 15]	.48
2018	37	23	−10 (−609; 236)	[−31; 2]	.11
2019	15	26	1 (−296; 195)	[−10;12]	.82
2020	11	10	0 (−177; 594)	[−6; 15]	.99

a Hodges–Lehmann method (a negative difference indicates that the group with at least one female FLA received fewer citations than the male-only FLA group).

b Mann–Whitney two-sided test.

Between 2017 and 2020, the NIH awarded 1472 new grants for research on kidney disease. Among these, 776 grants (52.7%) were allocated to just 40 academic medical centers (Supplementary Material S4). Across these 40 institutions, 475 (61.2%) grants were awarded to male PIs totaling US${\$}$193 787 257 in funds, while 301 grants (38.8%) were awarded to female PIs, totaling US${\$}$108 102 109 (Table 2). The disparity in NIH funding size between female and male PIs across all grant types was statistically significant, in favor of male PIs [95% CI: (−89 812; −24 258); *P* < .001]. However, this disparity was not seen across the 10 most frequently awarded grant types (*P* > .05 for all).

**Table 2:** Gender differences in National Institute of Health new grant allocations to PIs for research on kidney disease from 2017 to 2020. Only the 40 highest-funded academic medical centers are considered.

		Total funds (in USD)		Median funds (in USD)				
	Number of grants (% female PIs)	Male PIs	Female PIs	Male PIs	Female PIs	Median difference in funds (range)^a^	95% Confidence interval^b^	*P* value^b^
All grant types	776 (38.8)	193 787 257	108 102 109	340 545	222 038	−55 545 (−4 922 240; 5 497 815)	[−89 812; −24 258]	<.001
Stratification across the 10 most awarded grant types								
R01	302 (32.1)	108 629 476	48 919 757	467 726	485 481	2 500 (−4 876 974; 783 280)	[−35 889; 43 214}	.87
R21	73 (45.2)	8 924 627	8 000 754	233 151	227 120	−4 435 (−184 796; 593 625)	[−19 438; 15 750]	.54
F32	38 (39.5)	1 498 882	1 022 308	65 310	64 926	384 (−30 364; 69 796)	[−6 120; 8 564]	.82
K23	35 (54.3)	2 945 951	3 637 686	190 102	186 258	−448 (−45 066; 192 597)	[−12 061; 11 373]	.93
F31	35 (65.7)	524 258	924 390	36 375.5	44 044	1 869 (−88 613; 16 417)	[−2 002; 8 296]	.33
K08	35 (45.7)	3 184 534	4 177 344	168 264	168 090	−548.5 (−58 247; 1 555 983)	[−6 880; 5 849]	.90
U01	34 (32.35)	23 870 429	19 231 095	747 860	621 878	118 211 (−2 600 430; 5 497 815)	[−384 365; 1 863 213]	.56
R03	29 (41.4)	1 632 126	1 119 577	89 599	109 780.5	0 (−155 270; 152 822)	[−17 339; 28 686}	1
K01	24 (70.8)	981 683	2 455 165	153 791	150 526	1 750 (−69 604; 51 769)	[−8 547; 28 353]	.66
U19	18 (44.4)	45,41 008	2 042 884	483 287	236 980.5	−131 297.5 (−1 010 046; 697 509)	[−518 296; 116 389]	.31
Stratification by year								
2017	215 (35.3)	56 788 232	26 504 345	334 125	289 076.5	−9 558 (−4 921 490; 1 577 967)	[−75 215; 55 816]	.75
2018	172 (45,3)	34 972 761	22 957 072	327 427	204 065	−72 759.5 (−1 175 073; 2 428 452)	[−146 719; −9 415]	.02
2019	177 (36.7)	47 909 844	2,4086 374	345 913.5	172 852	−63 329.5 (−2 852 614; 2 853 288)	[−147 268; 0]	.04
2020	212 (38.7)	54 116 420	34 554 318	366 025	221 644	−71 653 (−2 329 862; 5 465 938)	[−138 417; −12 800]	.01

a Hodges–Lehmann method (a negative median value indicates that women were allocated less funding compared to men).

b Mann–Whitney two-sided test.

Our work is the first to show that male and female researchers in nephrology now exhibit relatively comparable academic performance and influence in terms of publications and citations. These results are encouraging, especially considering the known workforce imbalance favoring males within the field of nephrology in the USA. Furthermore, our research shows that women are last authors on ∼50% of manuscripts when there is “at least one female FLA,” suggesting that they are increasingly assuming the role of senior mentor rather than remaining in the mentee role. This finding is particularly positive given that statistics by the Association of American Medical Colleges clearly indicate that women in medicine are less likely to be promoted to associate or full professor [2]. By contrast, previous studies have shown increased female representation only in first authorship in high-ranking nephrology journals [4] without achieving equity in randomized controlled trials in nephrology [5, 6].

On the other hand, our findings demonstrate persistent imbalances in the size of NIH funding, consistent with previous studies [7]. Addressing disparities in funding allocation based on gender is crucial, as they can exacerbate attrition rates and perpetuate a vicious cycle of inequities in research opportunities and resources.

Our research faced several limitations. First, it relies on social perception of gender, rather than self-reported gender, although the two can be different. Additionally, despite best efforts in cohort selection, inherent biases remain as only research presented at ASN was considered, with only three subspecialties included and a limited number of years covered. Therefore, the results may not be generalizable to the entire nephrology community. Other limitations include possible unmeasured confounding factors such as including non-nephrologist authors, missing nephrology grants that are not under the spending category of “kidney disease,” and the absence of information on grant applications that were submitted and rejected.

## Supplementary Material

sfaf019_Supplemental_File
