# Supplementary material for: Women in academic nephrology: have we bridged the gender gap?
Source: Clin Kidney J. 2025 Jan 28;18(3):sfaf019. doi: 10.1093/ckj/sfaf019 (PMC11879463; doi:10.1093/ckj/sfaf019)
Supplement: sfaf019_Supplemental_File [file sfaf019_supplemental_file.docx]

**Supplementary material S1: Detailed Methods**

We reviewed all abstracts (N=1401) accepted for oral presentation at the American Society of Nephrology (ASN) annual Kidney Week meeting from 2017 until 2020 in order to capture the highest-impact nephrology research in recent years. Using Cochran’s formula, we determined that an ideal sample size of at least 302 abstracts would need to be examined to detect a meaningful difference in research performance between the two “first or last author” (FLA) gender groups (“male-only FLA” group and “at least 1 female FLA” group), assuming a margin of error of 0.05 and a 50% proportion of female authors. We therefore elected to focus on the 336 oral abstracts pertaining to glomerular disease (GN), onconephrology or transplant for manual screening.

Two independent investigators identified the gender of the FLA on each abstract and searched for corresponding articles in public databases (PubMed, Web of Science, and Google Scholar). In cases where gender determination was challenging, a comprehensive web search was conducted, and the gender was inferred from pronoun usage or photographs on institutional websites. We also examined the number of citations per article, with citation data recorded up to April 21st, 2024. Finally, we used the public NIH Principal Investigators (PI) database to analyze gender differences in new grant funds awarded in the field of nephrology between 2017 and 2020. Only grants allocated to the 40 highest-funded academic university hospitals were considered. Grants were identified by specifying the corresponding fiscal year, award type “new”, and NIH spending category “kidney disease”. For each grant, the type, amount, and gender of the PI were recorded.

The Chi-square contingency test was used to assess correlation between categorical variables, and the independent samples t-test to compare means. The Hodges-Lehmann method was adopted to estimate the median difference between two groups, and the Mann-Whitney 2-sided test to determine if this difference was statistically significant (significance threshold of p<0.05). All data analyses were performed using Python software (version 3.9.12).

**Supplementary table S2:** Distribution of abstracts chosen for oral presentation by gender groups, specialty, geographical location, year, and publication rate. GN: glomerular disease, FLA: first or last author.

| **Abstracts chosen for oral presentation** | **Male-only FLA** | **At least 1 female FLA** |
| --- | --- | --- |
| **Total, N (%)** | 170 (50.6) | 166 (49.4) |
| **By author type**  -Female first author  -Female last author  -Female first and last author | -  -  - | 86 (51.8)  36 (21.7)  44 (26.5) |
| **By specialty, N (%)**  -Transplant  -GN  -Onconephrology | 56 (16.7)  105 (31.2)  9 (2.7) | 68 (20.2)  87 (25.9)  11 (3.3) |
| **By geographical location, N (%)**  -North America  -Europe  -Asia  -Australia  -South America  -International | 82 (24.4)  45 (13.4)  19 (5.7)  3 (0.9)  0  21 (6.2) | 98 (29.2)  39 (11.6)  11 (3.3)  3 (0.9)  1 (0.3)  14 (4.2) |
| **By year, N (%)**  -2017  -2018  -2019  -2020 | 43 (12.8)  51 (15.2)  46 (13.7)  30 (8.9) | 43 (12.8)  44 (13.1)  50 (14.9)  29 (8.6) |
| **Corresponding published articles, N (% of overall)**  -overall  -with male first or last author on full paper  -with female first or last author on full paper | 129 (75.9)  115 (89.1)  14 (10.9) | 140 (84.9)  12 (8.6)  128 (91.4) |

**Supplementary table S3:** Difference in average number of abstracts over 4 years between the 2 FLA gender groups, stratified by sub-specialty (using Student’s t-test). GN: glomerular disease, FLA: first or last author. A p-value <0.05 is considered statistically significant.

|  | **Transplant** | **GN** | **Onconephrology** |
| --- | --- | --- | --- |
| Same FLA gender group on abstract and the article | 0.39 | 0.76 | 0.34 |
| Opposite FLA gender groups on abstract and article | 0.89 | 0.92 | 0.44 |
| Abstract with no corresponding article | 0.27 | 0.78 | 0.25 |

**Supplementary material S4:** Top 40 university medical centers receiving the highest NIH grant funding for new projects between 2017 and 2020 (by alphabetical order).

-Beth Israel Deaconess Medical Center

-Weill Cornell University

-University of Kentucky

-Wake Forest University Health Science Center

-Brigham and Women’s Hospital

-Columbia University Health Sciences

-Duke University

-Emory University

-Icahn School of Medicine at Mount Sinai

-Indiana University- Purdue

-Johns Hopkins University

-Massachusetts General Hospital

-Mayo Clinic Rochester

-New York university School of Medicine

-Northwestern University at Chicago

-Stanford University

-University of Alabama at Birmingham

-University of California Los Angeles

-University of California San Diego

-University of California San Francisco

-University of Colorado Denver

-University of Michigan at Ann Arbor

-University of Minnesota

-University of Pennsylvania

-University of Pittsburgh

-University of Washington

-UT Southwestern Medical Center

-Vanderbilt University Medical Center

-Washington University

-Yale University

-University of North Carolina at Chapel Hill

-University of Florida

-Children’s hospital of Philadelphia

-University of Kansas Medical Center

-University of Maryland Baltimore

-University of Mississippi Medical Center

-University of Southern California

-University of Tennessee Health Science Center

-Boston Medical Center

-University of Utah

-University of Virginia
